# The Dominance of Attentional Focus in Sitting Postural Control Among Patients with Stroke and the Characteristics of the Disconnectome

**DOI:** 10.3390/jcm14238515

**Published:** 2025-11-30

**Authors:** Shun Sawai, Shin Murata, Ken Ito, Shoya Fujikawa, Ryosuke Yamamoto, Yusuke Shizuka, Naoki Shimizu, Takato Nishida, Hideki Nakano

**Affiliations:** 1 Graduate School of Health Sciences, Kyoto Tachibana University, 34 Yamada-cho, Oyake, Yamashina-ku, Kyoto-shi 607-8175, Kyoto, Japan; sawai.neuroreha@gmail.com (S.S.); murata-s@tachibana-u.ac.jp (S.M.); fujikawa.pt@gmail.com (S.F.); r.yamamoto.pt@gmail.com (R.Y.); shizuka.reha@gmail.com (Y.S.); naokifd1419@gmail.com (N.S.); tnishida@seijoh-u.ac.jp (T.N.); 2 Japan Society for the Promotion of Science, Kojimachi Business Center Building, 5-3-1 Kojimachi, Chiyoda-ku 102-0083, Tokyo, Japan; 3 Department of Rehabilitation, Nagashima Neurosurgery Rehabilitation Clinic, 1st and 2nd floor Niitaka Clinic Center Building, 2-3-2 Niitaka, Yodogawa-ku, Osaka-shi 532-0033, Osaka, Japan; 4 Department of Physical Therapy, Faculty of Health Sciences, Kyoto Tachibana University, 34 Yamaca-cho, Oyake, Yamasina-ku, Kyoto-shi 607-8175, Kyoto, Japan; 110kmpt@gmail.com; 5 Department of Rehabilitation, Iwasa Hospital-Iwasa Maternity, 1-7-1 Yashiro, Gifu-shi 502-0812, Gifu, Japan; 6 Department of Rehabilitation, Kyoto Kuno Hospital, 22-500 Honmachi, Higashiyama-ku, Kyoto-shi 605-0981, Kyoto, Japan; 7 Department of Rehabilitation, Tesseikai Neurosurgical Hospital, 28-1 Nakanohon-Machi, Shijonawate-shi 575-8511, Osaka, Japan; 8 Faculty of Rehabilitation and Care, Seijoh University, 2-172 Fukinodai, Tokai-shi 476-0014, Aichi, Japan

**Keywords:** attentional focus, dominance, sitting postural control, stroke, disconnectome

## Abstract

**Background/Objectives**: Attentional focus, wherein motor performance differs between internal focus (IF), which directs attention to body parts, and external focus (EF), which directs attention to the external environment, has exhibited a dominance that enhances performance in healthy participants, whereby IF-dominant and EF-dominant groups show higher performance under IF and EF conditions, respectively. In this cross-sectional study, we aimed to verify the dominance of attentional focus in sitting postural control among patients with stroke and explored the involvement of the disconnectome in the dominance of attentional focus. **Methods**: Stroke survivors performed sitting postural control tasks under IF and EF conditions to evaluate attentional focus dominance. The severity of white matter disconnection was calculated using brain imaging analysis and compared between the IF-dominant and EF-dominant groups. **Results**: The results showed a dominance of attentional focus in sitting postural control in patients with stroke. Performance in the IF condition influenced the dominance of attentional focus. The EF-dominant group exhibited a higher disconnection severity in the corticostriatal tract (posterior). **Conclusions**: This study highlights the importance of customized interventions based on the dominance of attentional focus to improve sitting postural control abilities in patients with stroke.

## 1. Introduction

Among noncommunicable diseases, stroke is the second leading cause of death and the third leading cause of disability, with an estimated annual cost greater than 890 billion dollars. From 1990 to 2021, the number of stroke deaths, strokes, and people living with stroke has increased by 44%, 70%, and 86%, respectively [1]. This indicates a considerable increase in not only stroke deaths but also the number of stroke survivors. Interventions, including rehabilitation, after a stroke are crucial for minimizing motor and cognitive impairments and enabling individuals to regain independence in daily living.

Approximately 83% of patients with stroke exhibit impaired postural control [2], which affects walking independence at discharge [3] and independence in activities of daily living [4]. This underscores the importance of improving postural control in patients recovering from a stroke. In particular, impaired sitting postural control not only affects basic daily activities, such as eating and dressing, but also hinders higher-level motor functions, such as standing and walking, for which it is a prerequisite. Consequently, sitting postural control is considered a key indicator in stroke rehabilitation [5]. Therefore, methods to effectively improve sitting postural control in patients with stroke are urgently needed.

Depending on the target of attention, both motor performance and learning vary. This phenomenon of attentional focus consists of two types of attention: internal focus (IF), which directs attention to body parts during movement, and external focus (EF), which directs attention toward the external environment [6]. Traditionally, EF conditions have been reported to promote better performance and motor learning than IF conditions [7], and several studies have confirmed these effects in postural control [8]. The constrained action hypothesis has been proposed as the underlying mechanism that explains why EF conditions promote better performance and motor learning effects than IF conditions. It proposes that under IF conditions, performing movements while focusing attention on body parts induces conscious motor control, which disrupts automatic movement processes and consequently impairs performance and motor-learning [9]. However, recent studies have shown that EF conditions do not necessarily improve performance more than IF conditions. Instead, individuals tend to perform better under the attentional focus condition that aligns with their own dominance. This suggests the existence of an IF-dominant group that performs better in the IF, rather than the EF condition, and an EF-dominant group that performs better in the EF, rather than in the IF condition [7]. The dominance of attentional focus has been confirmed in upper limb movements, in healthy young adults [10,11,12,13], and in postural control during standing, in both healthy young adults [14,15] and older adults [16]. The factors that are associated with the dominance of attentional focus include motor imagery, sensory processing, and brain activity. Specifically, participants with vivid kinesthetic motor imagery showed higher motor-learning effects under the IF condition, whereas those with vivid visual-motor imagery showed higher effects under the EF condition [10]. Furthermore, the IF-dominant group exhibited greater responsiveness to superficial sensory input, whereas the EF-dominant group showed greater responsiveness to visual input, and this indicated differences in sensory processing characteristics [13]. The IF-dominant group showed higher activity in the parietal region, which is involved in superficial sensory processing, whereas the EF-dominant group showed higher activity in the frontal region, which is involved in error detection based on visual information, and these indicate differences in brain activity [14]. Thus, in healthy individuals, the dominance of attentional focus and its related factors has become clearer. In patients with stroke, upper limb motor control [17,18] and postural control [19,20] improved under EF, compared with IF, conditions in some studies, whereas other studies reported a higher performance under IF, rather than EF, conditions [21]. Furthermore, some studies found no difference in motor function improvement between the IF and EF conditions [22]. Therefore, the effects of attentional focus may not be uniform in patients with stroke. Additionally, patients with stroke exhibit preferences for optimal attentional focus conditions during upper limb movement, and characteristics of motor imagery ability are involved, similar to those in healthy individuals [10]. Thus, in patients with stroke, investigations on attentional focus and dominance are being conducted. However, the dominance of attentional focus in sitting postural control among patients with stroke has not been verified, and its relationship with the disconnectome of white matter fibers caused by stroke remains unclear.

Therefore, to generate hypotheses regarding the relationship between attentional focus dominance and disconnectome in sitting postural control among patients with stroke, this study (i) investigated the dominance of attentional focus in sitting postural control and (ii) used disconnectome analysis to explore differences in the disconnection severity between the IF-dominant and EF-dominant groups. Clarifying this may effectively facilitate improvements in sitting postural control in patients with stroke. This study hypothesized the presence of the dominance of attentional focus in patients with stroke, similar to that in young adults [14] and community-dwelling older adults [16].

## 2. Materials and Methods

### 2.1. Study Protocol

**Figure 1:**
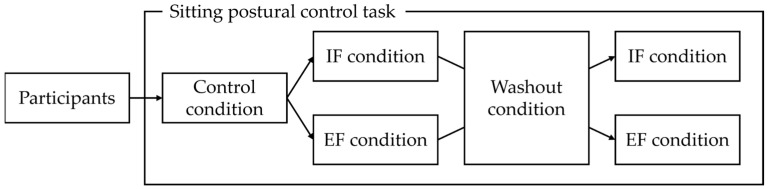
Study protocol. Participants performed a sitting postural control task under control conditions, followed by tasks under IF and EF conditions in random order. A washout condition was implemented between the IF and EF conditions. IF: internal focus, EF: external focus.

A randomized crossover design was used in this study. The participants first performed a sitting postural control task under the control condition, followed by tasks under the IF and EF conditions in a randomized order. A washout condition, identical to the control condition, was included between the IF and EF tasks (Figure 1).

### 2.2. Participants

This study included 25 patients (13 males and 12 females) with stroke who were either outpatients or inpatients at Iwasa Hospital-Iwasa Maternity, evaluated between May 2025 and September 2025. The inclusion criteria included (i) experience of a stroke; (ii) ability to maintain an upright sitting position without assistance or support for their upper limbs; (iii) no or only mild higher brain dysfunction (e.g., aphasia) and the ability to understand verbal instructions for this study; and (iv) a Mini-Mental State Examination score ≥ 21 [23,24], indicating no significant cognitive impairment. All participants provided informed consent, and the study was conducted in accordance with the Declaration of Helsinki. This study was approved by the local institutional ethics committee of Kyoto Tachibana University (approval no. 24–78).

### 2.3. Sample Size Calculation

The required sample size was calculated using G*Power software (G*Power 3.1; Heinrich Heine University, Düsseldorf, Germany) [25] for a two-factor analysis of variance with an effect size of 0.3, α = 0.05, and power (1 − β) = 0.8, whereby a sample size of 24 participants was required. Additionally, for the single regression analysis, calculations were performed with an effect size f^2^ = 0.35 (large), α = 0.05, and power (1 − β) = 0.8, indicating that a sample size of 25 participants was required.

### 2.4. Sitting Postural Control Task

**Figure 2:**
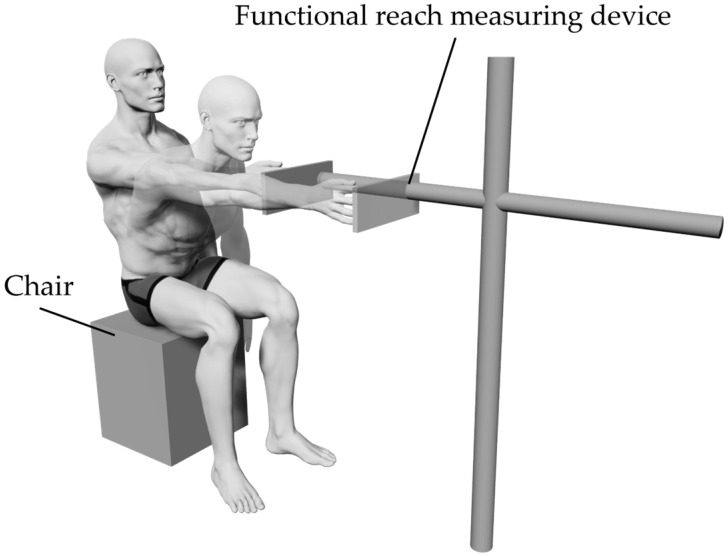
Experimental setup. Participants sat in a chair and reached forward with their non-paralyzed upper limb from a position of 90° flexion. The reach distance was measured using a functional reach measuring device.

In this study, the forward reach task while sitting was implemented as a sitting postural control task. Participants sat on a chair with their nonparalyzed hip, knee, and ankle joints flexed at 90°. They then reached forward from a position in which their non-paralyzed shoulder joint was flexed at 90°. A functional reach measuring device (TB-1256; Takada Bed Seisakusho, Osaka, Japan) was placed in front of each participant (Figure 2). The forward reach distance was measured by pressing the plate portion of the device with the fingertips on the non-paralyzed side. The participants were instructed to reach while looking at the device plate to ensure that the paralyzed upper limb was not supported by the body or chair, as well as to prevent truncal rotation.

A sitting postural control task was performed under controlled IF, EF, and washout conditions. In the control condition, participants received the verbal instruction, “Lean forward as much as possible,” without mention of where to direct their attention. In the IF condition, participants received the verbal instruction, “Focus your attention on your hand and reach it forward as far as possible,” which directed attention to the hand. In contrast, the EF condition used the verbal instruction, “Focus your attention on the front board and push it as far away as possible,” which directed attention to the board of the functional reach measuring device [26]. In the washout condition, participants received the same verbal instructions as in the control condition, and no reference was made to the target of attention. Consequently, a 5 min interval was provided between the first and second attentional focus conditions. Immediately after the IF and EF tasks, participants used a 101-point numerical rating scale (score range: 0–100) to rate the degree of attention that was directed toward the hand or board. Participants with a score < 60 were considered not to have directed attention as instructed by the verbal instructions [14]; however, no participants met this exclusion criterion.

Participants whose forward reach distance was longer in the IF condition than that in the EF condition were defined as the IF-dominant group, whereas those whose forward reach distance was longer in the EF condition than that in the IF condition were defined as the EF-dominant group [14]. Additionally, the difference in the sitting forward reach (EF condition − IF condition) was calculated as an indicator of attentional focus dominance. This metric indicates that a higher value signifies better performance in the EF condition than in the IF condition, whereas a lower value indicates better performance in the IF condition than in the EF condition.

### 2.5. Brain Images and Lesion Mapping

Brain images were analyzed using magnetic resonance imaging (MRI) or computed tomography (CT) scans that were acquired in clinical settings. The most recent images available for each patient were analyzed. For patients with both MRI and CT scans, only the MRI scan was selected. Consequently, MRI and CT were used for analysis in 20 and 5 participants, respectively. The time from stroke onset to MRI or CT acquisition had a median value of 27 days (minimum–maximum: 0–637 days).

Using MRIcroGL, lesion mapping was performed manually by two researchers with extensive knowledge and experience in stroke and lesion mapping [27]. Lesions were identified using diffusion-weighted, T2-weighted, fluid-attenuated inversion recovery (FLAIR), and CT images, and then mapped onto FLAIR or CT images. Next, the Clinical Toolbox [28] of SPM12 [29] was used to normalize the FLAIR or CT images and lesion maps to Montreal Neurological Institute (MNI) space. This conversion transformed the normalized data into a voxel size of 1 mm × 1 mm × 1 mm. Additionally, the normalized lesion maps were inverted to unify the lesions on the right side [30].

### 2.6. Disconnectome Analysis

Disconnectome analysis was performed using the Lesion Quantification Toolkit (LQT) [31], which incorporates a large-scale normative connectome atlas. Lesions were embedded in streamlines from the Human Connectome Project-1065 Atlas [32] to generate voxel-based disconnectome maps. Then, using FMRIB Software Library (FSL) version 6.0.7.16, a widely used MRI analysis software package [33], the disconnectome maps for each patient were integrated to create average disconnectome maps for the IF-dominant and EF-dominant groups.

### 2.7. Statistical Analysis

The Shapiro–Wilk test was performed on all data to confirm normality. Next, for variables that showed a normal distribution, we compared participant characteristics between the IF-dominant and EF-dominant groups using Fisher’s exact test and independent *t*-tests for categorical and continuous variables, respectively. The Mann–Whitney *U* test was used for variables that did not show a normal distribution. In addition, a repeated-measures analysis of variance was conducted to examine potential order effects, using two factors: order (first attentional focus condition, second attentional focus condition) and attentional focus dominance (IF-dominant group, EF-dominant group). For factors with significant main effects or interactions, Bonferroni post hoc tests were performed. Furthermore, a simple regression analysis was performed with the difference in sitting forward reach (EF condition − IF condition) as the dependent variable, and the forward reach distance in each condition as the explanatory variable. The disconnection severity calculated by the LQT was compared between the IF-dominant and EF-dominant groups using the Mann–Whitney *U* test. As this comparison of disconnection severity was an exploratory analysis aimed at hypothesis generation, multiple comparisons were not adjusted [34].

All statistical analyses were performed using SPSS version 30.0 (IBM Corp., Armonk, NY, USA), with the threshold of statistical significance set at 5%.

## 3. Results

**Figure 3:**
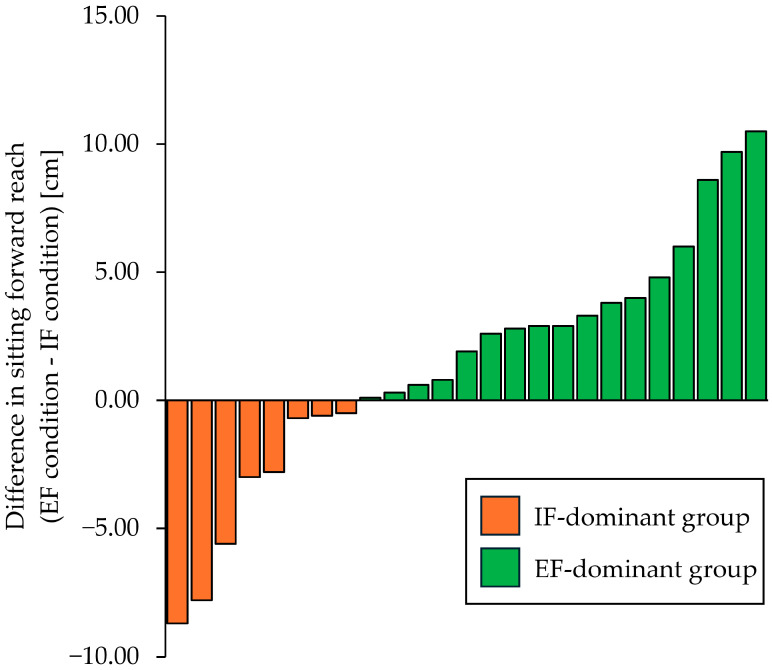
The dominance of attentional focus. The vertical axis shows the difference in sitting forward reach (EF condition − IF condition), with higher values indicating a longer reach distance in the EF condition compared to the IF condition. The orange bars represent the IF-dominant group, which showed a negative difference in the sitting forward reach, whereas the green bars represent the EF-dominant group, which showed a positive difference in the sitting forward reach. Participants in this study were divided into the IF- and EF-dominant groups. IF: internal focus, EF: external focus.

First, by comparing forward reach under IF and EF conditions, participants were divided into an IF-dominant group, wherein the forward reach was longer under IF than EF conditions, and an EF-dominant group, wherein the forward reach was longer under EF than under IF conditions (Figure 3). When comparing the participants’ basic information, no significant intergroup differences were found between the IF-dominant and EF-dominant groups for any variable (*p* > 0.05; Table 1).

Values are expressed as the mean ± standard deviation for continuous variables and number [*n*] for categorical variables. An independent *t*-test was performed for normally distributed continuous variables (e.g., age, height, and body weight). For non-normally distributed continuous variables (duration from stroke onset and the MMSE score), the Mann–Whitney *U* test was performed. Fisher’s exact test was performed for categorical variables (e.g., sex, stroke type, and lesioned hemisphere). MMSE, Mini-Mental State Examination; IF, internal focus; EF, external focus.

**Figure 4:**
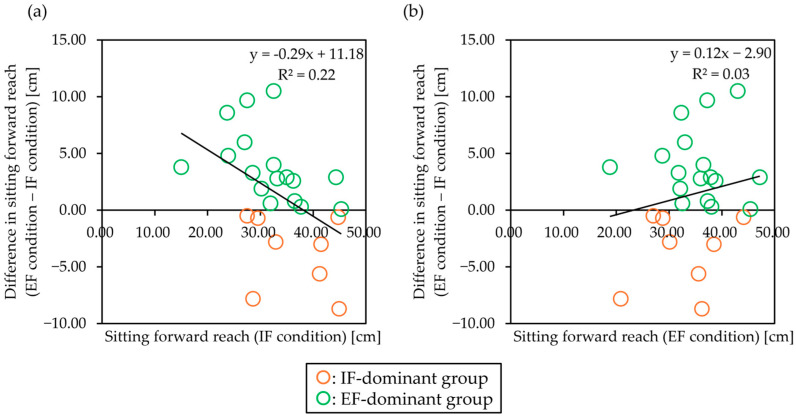
Effects of performance in IF and EF conditions on the dominance of attentional focus. The orange plots represent the IF-dominant group, and the green plots represent the EF-dominant group. (**a**) The scatterplot shows the relationship between the difference in sitting forward reach (EF condition − IF condition) and sitting forward reach (IF condition). The simple regression model showed that the difference between the two variables was significant (*p* = 0.01). (**b**) The scatterplot shows the relationship between the difference in the sitting forward reach (EF condition − IF condition) and the sitting forward reach (EF condition). The simple regression model between the two variables was not significant (*p* = 0.39). IF: internal focus, EF: external focus.

To examine the order effect of the tasks, the first and second attentional focus conditions were compared. The analysis showed no significant order × group interaction (F = 0.61, *p* = 0.44, partial η^2^ = 0.03), no significant main effect of order (F = 1.28, *p* = 0.27, partial η^2^ = 0.05), and no significant main effect of group (F = 0.06, *p* = 0.81, partial η^2^ < 0.01). In addition, a simple regression analysis was conducted with the difference in sitting forward reach (EF condition − IF condition) as the dependent variable and the sitting forward reach in each condition as the explanatory variable. The results showed a significant relationship between the difference in the sitting forward reach (EF condition − IF condition) and the sitting forward reach (IF condition) (B = −0.29, SE = 0.12, β = −0.47, *p* = 0.02, R^2^ = 0.22; Figure 4a). In contrast, no significant relationship between the difference in the sitting forward reach (EF condition − IF condition) and the sitting forward reach (EF condition) (B = 0.13, SE = 0.14, β = 0.18, *p* = 0.39, R^2^ = 0.03; Figure 4b) was noted.

**Figure 5:**
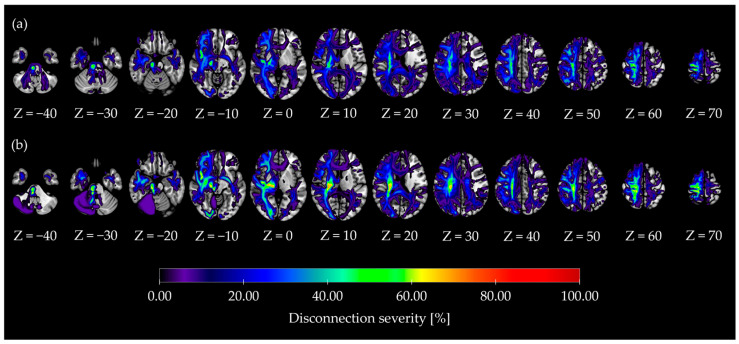
Disconnectome map in IF- and EF-dominant groups. Below each slice, the normalized vertical Z-coordinate in MNI space is shown. The color scale represents disconnection severity per voxel. (**a**) Disconnectome map in the IF-dominant group. (**b**) Disconnectome map in the EF-dominant group.

**Figure 6:**
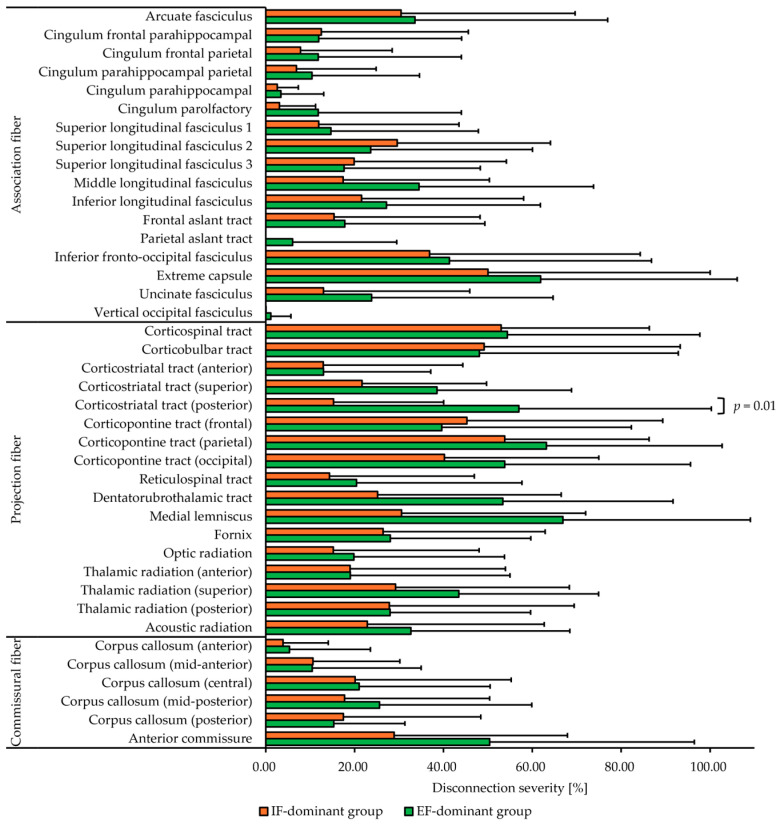
Comparison of the disconnection severity between IF-dominant and EF-dominant groups. In this bar graph, the horizontal axis represents disconnection severity. The data are shown as the mean and standard deviation. Disconnection severity in the corticostriatal tract (posterior) was significantly higher in the EF-dominant group compared to the IF-dominant group (*p* = 0.01). IF: internal focus, EF: external focus.

Disconnectome analysis revealed damaged white matter tracts in the IF-dominant (Figure 5a) and EF-dominant (Figure 5b) groups. In addition, when comparing the disconnection severity between the groups for each white matter tract, the disconnection severity in the posterior corticostriatal tract (posterior) was significantly higher in the EF-dominant group than in the IF-dominant group (U = 26.00, *p* = 0.01, rank-biserial r = 0.62). However, no significant group differences were observed in disconnection severity for the other white matter tracts (*p* > 0.05; Figure 6).

## 4. Discussion

This study investigated the dominance of attentional focus in sitting postural control in patients with stroke. Additionally, using brain images from the IF-dominant and EF-dominant groups, disconnectome analysis was performed to explore the characteristics of white matter fiber damage in each group by comparing disconnection severity. Accordingly, the participants were divided into IF-dominant and EF-dominant groups. Furthermore, the IF condition of sitting postural control performance influenced the dominance of the attentional focus. Additionally, compared with the IF-dominant group, the EF-dominant group exhibited higher disconnection severity in the corticostriatal tract (posterior). These results suggest that the conditions for improving sitting postural control vary among individual patients with stroke and that the dominance of attentional focus is observed.

### 4.1. The Dominance of Attentional Focus in Patients with Stroke

In this study, the participants were divided into IF-dominant and EF-dominant groups based on the difference in sitting forward reach distance between the IF and EF conditions. This result indicates that, in sitting postural control of patients with a stroke, interindividual differences exist in the condition that improves performance. Many studies on attentional focus have reported higher performance and motor learning effects in the EF condition than in the IF condition, and similar effects have been reported for postural control [8]. In contrast, recent studies have revealed interindividual variations, including among healthy young adults and older participants, in the optimal conditions for improving postural control [14,15,16]. In this study, the participants were divided into the IF-dominant and EF-dominant groups to reveal the dominance of attentional focus. Thus, our study’s results support recent research on inter-individual differences in the optimal conditions for improving performance.

No evidence of an order effect was observed in the sitting forward reach task, indicating that task order did not influence performance. In contrast, the regression analysis using the difference in sitting forward reach (EF condition − IF condition) revealed that only performance under the IF condition significantly predicted the difference between EF and IF conditions, whereas performance under the EF condition did not. These results suggest that performance under the IF condition may play an important role in the dominance of attentional focus. In other words, individuals who can more effectively exert conscious postural control under the IF condition may be more likely to show IF dominance. This finding is consistent with the constrained action hypothesis [9], which proposes that attention directed toward body movements can alter the balance between conscious and automatic control. Therefore, participants with high sitting postural control performance in the IF condition belonged to the IF-dominant group, whereas those with low sitting postural control performance in the IF condition belonged to the EF-dominant group. Regarding attentional focus, it has been proposed that the IF condition promotes conscious movement control, whereas the EF condition promotes automatic movement control [9]. IF-induced conscious control leads to excessive control attempts that thereby reduce motor performance [9]. In contrast, studies on attentional focus in patients with stroke reported conflicting results—some showed improved motor performance in the EF condition [18,19,20,21], whereas others reported improved performance in the IF condition [22]. The reason IF conditions improve motor performance in patients with stroke is that these patients tend to consciously control their movements in daily life [35], and IF conditions contribute to performance improvement by promoting the conscious control of appropriate movements that the patients habitually perform. However, the effect of conscious control via IF on motor performance in stroke patients may not be uniform. Therefore, 0it is possible that some participants in the IF condition exerted excessive conscious control, whereas others imposed appropriate conscious control over functions that they habitually performed. This suggests that the performance in the IF condition is influenced by the dominance of the attentional focus. However, the sitting postural control performance in the EF condition did not significantly influence attentional focus dominance. A study examining the changes in balance ability under IF and EF conditions in patients with stroke reported no significant differences between the conditions. However, it found that EF was effective for patients with relatively good balance and sensory function but lower attention function [36]. Thus, although the performance under the EF condition remains relatively consistent regardless of the patient’s physical and cognitive function, the performance under the IF condition varies depending on the specific patient’s functional characteristics. Furthermore, the EF condition required fewer attentional resources than the IF condition [37]. Considering these findings, although performance in the EF condition was relatively maintained, performance in the IF condition may have varied depending on the participant’s characteristics. Therefore, in this study, although performance in the IF condition was influenced by the dominance of attentional focus, performance in the EF condition was maintained regardless of the dominance of attentional focus and may not have been affected by it.

### 4.2. Differences in Disconnection Severity Between the IF-Dominant and EF-Dominant Groups

This study compared the disconnection severity between the IF-dominant and EF-dominant groups and explored the involvement of the white matter tracts in the dominance of attentional focus. Compared to the IF-dominant group, the EF-dominant group exhibited significantly higher disconnection severity in the corticostriatal tract (posterior). The corticostriatal tract is a broad white matter fiber bundle that connects the cortex and striatum; the anterior part projects from the frontal association cortex to the caudate head and anterior putamen, the superior part projects from the sensorimotor cortex to the putamen, and the posterior part projects from the sensorimotor cortex to the posterior putamen [38,39]. Specifically, the posterior corticostriatal tract forms a part of the motor loop within the corticobasal ganglia loop, which contributes to motor execution and sensorimotor integration [40]. Therefore, the EF-dominant group may have experienced a higher degree of damage to the posterior corticostriatal tract. Owing to impaired sensorimotor integration, the patients in the EF-dominant group may have excessively controlled their movements during conscious sitting postural control under IF conditions, which could have resulted in lower performance. These findings provide preliminary evidence that disconnection severity may be involved in the dominance of attentional focus in sitting postural control among patients with stroke. However, it is important to note that these findings remain hypothesis-generating and do not provide grounds for drawing causal conclusions.

### 4.3. Limitations

This study had some limitations. First, this study was unable to assess task-independent factors, such as characteristics of motor imagery [10], that previous research has suggested may be related to attentional focus. Future research is recommended to examine task-independent factors in detail. Second, as the primary aim was to examine the dominance of attentional focus in sitting postural control among patients with stroke, we were unable to standardize factors, such as stroke type or hemispheric lesion. Consequently, although this study verified the differences in the dominance of attentional focus among patients with stroke, the standardization of patient characteristics could yield more detailed insights. Third, regarding the results of the disconnectome analysis in this study, the sample size was small, and an exploratory analysis was conducted for hypothesis generation. Therefore, caution is required when interpreting the results of intergroup comparisons of disconnection severity. Future studies should increase the sample size to verify the hypotheses of this study. Fourth, this study does not account for stroke severity, the associated length of hospital stays, or the number of days since stroke onset. Future research should incorporate analyses that consider stroke severity using standard stroke functional assessments such as the Fugl–Meyer Assessment. Fifth, in this study, participants were divided into two groups based on performance, following previous research [10,11,12,13,14,15,16,17]. However, some participants exhibited small differences in performance between the IF condition and the EF condition. Future research should clarify how participants with small performance differences between conditions should be classified.

## 5. Conclusions

This study investigated the dominance of attentional focus in sitting postural control among patients with stroke. Additionally, to explore the white matter disconnection associated with this dominance, the severity of disconnection was compared between the IF-dominant and EF-dominant groups. The results revealed interindividual differences in the conditions that enhanced sitting postural control performance. Furthermore, the performance in the IF condition, which promotes conscious control, predicts the dominance of attentional focus. These findings suggest the importance of customized interventions, based on the dominance of attentional focus, in improving sitting postural control in patients with stroke. Moreover, performance in the IF condition should be considered when developing customized interventions. Additionally, the disconnectome analysis, although exploratory, revealed a higher severity of disconnection in the corticostriatal tract (posterior), which was involved in sensorimotor integration within the EF-dominant group. Therefore, this preliminary finding should be considered hypothesis-generating as it does not provide sufficient evidence to draw definitive conclusions.

## Figures and Tables

**Table 1:** Characteristics of the study participants.

	All (*n* = 25)	IF-Dominant Group (*n* = 8)	EF-Dominant Group (*n* = 17)	*p*-Value
Age [years]	62.36 ± 11.37	68.88 ± 11.51	59.24 ± 10.63	0.05
Height [cm]	161.01 ± 9.30	156.84 ± 11.43	163.09 ± 8.05	0.13
Body weight [kg]	58.08 ± 11.53	54.50 ± 12.75	59.76 ± 11.28	0.31
Sex: male/female [*n*]	13/12	3/5	10/7	0.41
Stroke type: hemorrhagic/cerebral infarction [*n*]	14/11	3/5	11/6	0.39
Lesioned hemisphere: right/left [*n*]	9/16	3/5	6/11	1.00
Duration from stroke onset [days]	224.44 ± 316.38	169.75 ± 257.54	250.18 ± 352.42	0.71
MMSE [points]	27.08 ± 2.64	26.86 ± 3.31	27.18 ± 2.46	0.93

## Data Availability

The data presented in this study are available upon request from the corresponding author. The data is not publicly available due to privacy or ethical restrictions.

## Abbreviations

The following abbreviations are used in this manuscript:

CT	computed tomography
EF	external focus
FLAIR	fluid-attenuated inversion recovery
FSL	FMRIB Software Library
IF	internal focus
MNI	Montreal Neurological Institute
MMSE	Mini-Mental State Examination
MRI	magnetic resonance imaging
